# Apple Vision Pro: a new horizon in psychological research and therapy

**DOI:** 10.3389/fpsyg.2023.1280213

**Published:** 2023-11-02

**Authors:** Zhihui Zhang, Lluis Giménez Giménez Mateu, Josep M. Fort

**Affiliations:** Escola Tècnica Superior d' Arquitectura de Barcelona, Universitat Politècnica de Catalunya, Barcelona, Spain

**Keywords:** Apple Vision Pro, virtual reality, telepsychotherapy, emotion recognition, psychological experiments

Virtual Reality (VR) harbors immense potential for advancing psychological therapy and emotional research, despite presenting several challenges (Riva, 2022). The release of Apple Vision Pro has unveiled new opportunities in the realm of psychological emotion research, particularly with its facial expression system enabling facial emotion recognition within a virtual reality environment. We will elucidate the new perspectives Apple Vision Pro brings to Psychological Research and Therapy by delving into its Multi-Sensor Technology, High Resolution, and Remote Scene Meeting Capabilities.

## Multi-sensor advancements for VR facial emotion recognition

Measuring emotions in virtual reality (VR) has mainly involved using electroencephalogram (EEG) devices (Suhaimi et al., 2020; Pinilla et al., 2021). However, the conflict between the mobility associated with VR experiences and the stillness required for reliable EEG measurements casts doubts on the viability of EEG-based VR emotion measurement. The limitations of EEG-based emotion measurement in VR have led to the exploration of alternative methods for more reliable emotion recognition. One such alternative emerged with the introduction of facial recognition technologies, which promise a less obtrusive and more natural means of gauging emotional responses compared to EEG. The pivotal moment arrived with the debut of Fove 0 in 2017, which pioneered the incorporation of eye-tracking technology in VR, marking the onset of a new era in VR Emotion Recognition technology. The advent of eye-tracking provided a fresh perspective and an additional layer of data to better understand users' emotional responses within virtual environments. Since then, a plethora of devices, each with their distinct features, capabilities, and limitations for research applications, have emerged, as detailed in Table 1.

**Table 1:** Comparison of technical specifications and tracking capabilities of virtual reality devices.

**Release year**	**Name**	**Positional tracking**	**Resolution**	**Refresh rate**	**Tracking capability**
2017	Fove 0	Yes	1,280 × 1,440	70 Hz	Eye-tracking
2017	Deepoon VR E3	Yes (360° laser room-scale positioning)	1,280 × 1,440	70 H	Eye-tracking
2018	VRgineers XTAL	Yes (AR Tracking, Optitrack and Lighthouse)	2,560 × 1,440	70 Hz	Leap Motion hand-tracking, eye-tracking
2018	StarVR One	Inside-out markered (Lighthouse)	1,830 × 1,464	90 Hz	Tobii eye tracking
2019	Varjo VR-1	Yes	1,920 × 1,080	60 Hz	Eye-tracking
2021	Varjo Aero	Yes	2,880 × 2,720	90 Hz	Eye-tracking
2019	HTC Vive Cosmos	Yes	1,440 × 1,700	90 Hz	ye-tracking, Mouth tracking (VIVE Facial Tracker 1camera)
2021	Varjo VR-3	Yes	2,880 × 2,720	90 Hz	Eye-tracking
2022	Meta Quest Pro	Inside-out	1,800 × 1,920	90 Hz	Eye tracking, Face tracking, Mouth tracking (5 external (one color passthrough), 5 internal sensors)
2023	PlayStation VR2	Yes	2,000 × 2,040	90 Hz	Eye-tracking
2023	Apple Vision Pro	Inside-out	3,680 × 3,140	90 Hz	Eye tracking, Face tracking, Mouth tracking (12 cameras, 5 sensors)

However, the reliance on eye-tracking alone has shown intrinsic limitations, particularly in capturing the full spectrum of emotional and psychological reactions (Levitan et al., 2022). The intersection of facial recognition technology and VR is gaining traction, aiming to provide a more nuanced understanding of emotional responses within virtual realms. Devices like Meta Quest Pro attempted to integrate facial recognition technologies but faced technical and applicative constraints, limiting their utility in comprehensive emotion recognition research. In contrast, the 2023 launch of Apple Vision Pro represents a significant leap forward by

seamlessly combining high-fidelity sensor technology with facial recognition, unlocking new potential for emotion recognition in VR. Uniquely, Apple Vision Pro utilizes individualized facial scans of each user, as opposed to the generalized facial simulation technology employed by other notable devices like those from Meta and Vivo (Zhang et al., 2023). With an intricate array of 12 cameras and 5 sensors, Apple Vision Pro ensures nuanced tracking of facial expressions, presenting a detailed framework for emotion detection. This robust sensor framework not only ensures accurate and personalized facial recognition and expression transmission, marking a substantial advancement in VR facial emotion recognition.

## Enhanced realism for experimental and therapeutic applications

Traditional psychological experiments often grapple with the challenge of ecological validity due to the artificial laboratory settings. The Apple Vision Pro, boasting a superior resolution of 3680x3140, offers remarkable scene simulation capabilities, enabling more realistic and immersive environments for both experimental and therapeutic applications. This heightened realism, facilitated by the device's high-definition rendering, paves the way for more authentic and valid research outcomes. For instance, in exposure therapy, therapists can harness the Apple Vision Pro to simulate real-life scenarios in a controlled yet authentic setting, aiding patients in gradually confronting and overcoming their fears (Carlin et al., 1997; Krijn et al., 2004; Barrett et al., 2023). The enhanced resolution and field of view not only deepen users' immersion in highly detailed and broad virtual environments but also significantly improve the ecological validity of psychological and emotional research within VR, heralding a promising trajectory for future innovations and applications in VR-based emotional and behavioral studies.

## Potential of 3D rendering and facial expressions in telepsychotherapy

Telepsychotherapy has brought convenience to those unable to undergo face-to-face therapy. However, traditional methods like video calls fall short in terms of realism and immersive experience (Kocsis and Yellowlees, 2018; Poletti et al., 2020; Rosen et al., 2020). The introduction of Apple Vision Pro has revolutionized this domain. Its capability to render 3D scenes and characters, coupled with the realistic portrayal of facial expressions, offers immense potential in remote psychotherapy. Such detailed representation allows for a genuine, immersive therapeutic environment, providing a tangible interactive space for both therapists and patients. The realistic depiction of facial expressions, critical for psychotherapy, enables therapists to gauge patients' emotions and responses accurately, thus potentially enhancing the effectiveness of remote therapeutic interventions (Wiederhold and Wiederhold, 2009).

## Confronting ethical and operational challenges

Apple's products are known for their maturity and stability. The Apple Vision Pro, despite its high performance, comes with a hefty price tag, making it unaffordable for many. Additionally, the use of individualized facial scans presents two challenges: increased operational difficulty and time in conducting related research experiments, and privacy concerns compared to using virtual avatars. Addressing these issues while capitalizing on the technological advancements of VR emotion recognition remains a critical task for the industry.

## Conclusion

The Apple Vision Pro serves as a beacon of advancement in psychological research and therapy, integrating high-fidelity sensor technology and realistic 3D rendering. Looking ahead, further technological enhancements of Apple Vision Pro might include more precise emotion recognition algorithms and real-time data analytics, offering refined tools for understanding human emotions and behavior in virtual environments. The potential integration of these advancements could bolster telepsychotherapy and exposure therapy, among other therapeutic applications, providing more personalized and effective treatment solutions.

The technological prowess of Apple Vision Pro not only addresses current challenges in the field but also lays a foundation for future exploration. As the realms of technology and psychological sciences continue to merge, the potential for new, innovative methodologies in therapy and research expands. The Apple Vision Pro represents a significant stride toward a tech-driven era in psychological research and therapy, with its potential to unveil deeper insights into the human psyche and contribute to the betterment of mental health services worldwide.

## Author contributions

ZZ: Conceptualization, Writing—original draft, Writing—review & editing. JF: Methodology, Supervision, Writing—review & editing. LG: Methodology, Supervision, Writing—review & editing.
